# Rapid cognitive improvement in Alzheimer's disease following perispinal etanercept administration

**DOI:** 10.1186/1742-2094-5-2

**Published:** 2008-01-09

**Authors:** Edward L Tobinick, Hyman Gross

**Affiliations:** 1 Department of Medicine, Institute for Neurological Research, a private medical group, inc., Los Angeles, USA; 2 Department of Neurology, USC School of Medicine, Los Angeles, USA

## Abstract

Substantial basic science and clinical evidence suggests that excess tumor necrosis factor-alpha (TNF-alpha) is centrally involved in the pathogenesis of Alzheimer's disease. In addition to its pro-inflammatory functions, TNF-alpha has recently been recognized to be a gliotransmitter that regulates synaptic function in neural networks. TNF-alpha has also recently been shown to mediate the disruption in synaptic memory mechanisms, which is caused by beta-amyloid and beta-amyloid oligomers. The efficacy of etanercept, a biologic antagonist of TNF-alpha, delivered by perispinal administration, for treatment of Alzheimer's disease over a period of six months has been previously reported in a pilot study. This report details rapid cognitive improvement, beginning within minutes, using this same anti-TNF treatment modality, in a patient with late-onset Alzheimer's disease. Rapid cognitive improvement following perispinal etanercept may be related to amelioration of the effects of excess TNF-alpha on synaptic mechanisms in Alzheimer's disease and provides a promising area for additional investigation and therapeutic intervention.

## Background

Neuroinflammation with overexpression of cytokines is a standard characteristic of the brain pathology present in Alzheimer's disease [1-4]. Involvement of the pro-inflammatory cytokine tumor necrosis factor-alpha (TNF-alpha) in the pathogenesis of Alzheimer's disease has long been suspected [5-9]. Increasing basic science, genetic, and clinical evidence now supports the concept that excess TNF-alpha plays a central role in Alzheimer's disease [5-25].

In 1998 etanercept, a potent anti-TNF therapeutic, was approved for human use, with rheumatoid arthritis as the initial indication. Etanercept is a recombinant dimeric fusion protein consisting of the extracellular ligand-binding portions of two human p75 TNF-alpha receptors linked to the Fc fragment of human IgG1. Etanercept binds to TNF-alpha and blocks its interaction with cell surface TNF-alpha receptors, thereby reducing the biologic effect of excess TNF-alpha. The medical community now has more than 1 million patient-years of experience using etanercept for treatment of a variety of inflammatory disorders in which TNF-alpha has been postulated to play a role [26].

In 2006 the present authors published a pilot study which provided proof-of-concept that a novel method of administration of etanercept was efficacious for the treatment of Alzheimer's disease [20]. This novel method, perispinal extrathecal administration in the posterior neck, was hypothesized to improve delivery of etanercept to the brain via the cerebrospinal venous system[21,27]. In an open-label study of 15 patients treated weekly for a period of six months, perispinal extrathecal administration of etanercept led to sustained cognitive improvement in this cohort of patients with probable Alzheimer's disease ranging in severity from mild to severe [20]. The concept of TNF-alpha-inhibition for the treatment of Alzheimer's disease has been recently reviewed[22], and is supported by additional recent publications [16,18,23-25].

The author's 2006 pilot study was an IRB-approved clinical trial whose protocol included monthly cognitive testing. There was no provision for testing at shorter intervals. Nevertheless during the six-month duration of this clinical trial, and now for a period of more than three years, the authors have noted an unexpected and largely unprecedented clinical phenomenon, which they have observed on a weekly basis in the majority of the patients treated. This unexpected, but repeatedly observed phenomenon was a noticeable clinical improvement within minutes of perispinal etanercept administration[21]. Although this rapid clinical improvement could not be measured using the assessment intervals which were included in the original clinical trial protocol, the authors recently had the opportunity to quantitate this rapid treatment effect using several standardized cognitive tests in a new patient treated as part of their standard practice of medicine. This patient is the subject of the present case report.

Synaptic dysfunction has gained increasing recognition as an important pathophysiological component of Alzheimer's disease [28-32]. Recent evidence suggests that TNF-alpha may be involved in this synaptic dysfunction [33-37]. Although TNF-alpha is most widely known as a pro-inflammatory cytokine, basic science studies suggest that TNF-alpha may also have entirely different functions in the Alzheimer brain: as a regulator of synaptic transmission; and as a mediator of β-amyloid and β-amyloid oligomer disruption of memory mechanisms [33-37].

The present report details the clinical observations in this patient with late-onset Alzheimer's disease. It then attempts to provide a theoretical framework to explore the pathophysiological mechanisms underlying the rapid improvement in the patient's behavior and cognition, particularly those involving TNF-alpha and synaptic function, which may account for the rapid clinical effects observed after perispinal etanercept administration.

## Case presentation

### History of present illness

The patient is an 81 y.o. physician who was referred in October 2007 for consideration of perispinal etanercept treatment by his internist. The patient had been in good general health until about two years before presentation, at which time his wife noted progressive memory difficulties and difficulties with mathematical calculation.

His wife took over check writing, and the patient was evaluated by a neurologist 18 months ago. Donepezil was prescribed but was not tolerated and was discontinued after four months. Memantine and rivastigmine were prescribed but also were not tolerated by the patient. Galantamine was begun six months prior to presentation, and continued to the present at a dose of 8 mg per day, reduced from a maximum attempted dose of 16 mg per day due to side effects. No beneficial effect from the galantamine has been noted by the patient's family or his primary care physician.

The patient's current medications include atorvastatin, low dose aspirin, and galantamine 8 mg per day.

### Past medical history

The patient's past medical history include a lumbar discectomy and fusion performed twenty years previously, with chronic intermittent low back pain and spinal stenosis; hypercholesterolemia; and localized prostate cancer, successfully treated with localized radiation without sign of recurrence. Otherwise the past medical history was unremarkable, with no history of diabetes, stroke, transient ischemic attack, head trauma, epilepsy, loss of consciousness, hallucinations, recent infection, demyelinating disease, or blood or bleeding disorders, lymphoma, congestive heart failure, autoimmune disease, immunosuppression, tuberculosis or visual disturbance. Purified protein derivative testing was negative. Family history of dementia or neurodegenerative disease was negative.

### Physical examination

General physical exam was unremarkable. Blood pressure was 150/90, pulse 80 and regular, afebrile, respirations 12. The chest was clear to percussion and auscultation. There were no cardiac murmurs and no bruits in the neck.

### Neurologic examination one day prior to etanercept treatment

The results of neurological examination by author HG one day prior to etanercept administration were as follows:

The cranial nerve examination II-XII was within normal limits: the pupils were four mm, round, and reactive to light and accommodation. The discs were flat. The visual fields were full to confrontation; extraocular movements were conjugate and full without nystagmus. Facial motion and sensation were symmetrical and without deficits. The patient was able to hear finger rustling in both ears, however his auditory processing appeared to be decreased, and he intermittently asked for questions to be repeated. Weber was midline, air conduction was greater than bone conduction bilaterally, palate was normal, sternocleidomastoid strength was normal, trapezius strength was normal, and tongue movements were normal. Tremor was absent.

Motor and cerebellar examination was unremarkable. Deep tendon reflexes were as follows: biceps two plus and symmetrical, triceps one plus and symmetrical, supinator on the right absent, on the left one plus, quadriceps one plus bilaterally, ankles absent on the right and one plus on the left. Toe signs were flexor. There is no pronator drift, abnormal involuntary movement, or Romberg. There was symmetrical muscle tone, bulk, and development throughout. There was normal station and gait, and normal rapid alternating movement, normal finger to finger, and normal gait. There were no frontal release signs except for a positive bilateral palmomental sign.

Sensory examination responses were intact throughout to joint position, pinprick, and light touch. There was mild decrease in vibratory sense in the right hallux only.

Mental status examination performed one day prior to etanercept administration revealed a dignified appearing man who appeared younger than his stated age, neatly groomed and socially outgoing in a superficial engaging fashion. There was no agitation, suspiciousness, or hostility. He appeared at times to be inappropriately euphoric. He had difficulty recalling personal autobiographical information such as birthday or his father's occupation. He could not recall the names of any of the physicians who treated him. He was not oriented to the calendar date, day of the week, year, place, city, or state.

His spontaneous speech appeared fluent with normal prosody, articulation, and rate of speech production without any dysarthria. On detailed examination he had marked anomic aphasia, i.e., when shown the first ten pictures of the Boston Naming Test[38] the patient could not name nine out of ten, but used marked circumlocution. He was able to repeat complex sentences without difficulty. On a verbal fluency task, i.e., when asked to list all of the words that start with the letter F in 60 seconds he listed five words with five perseveratory responses and one neologism. When given a semantic task, i.e. asked to list all the animals he could in 60 seconds he could only list two, dog and cat.

**Figure 1:**
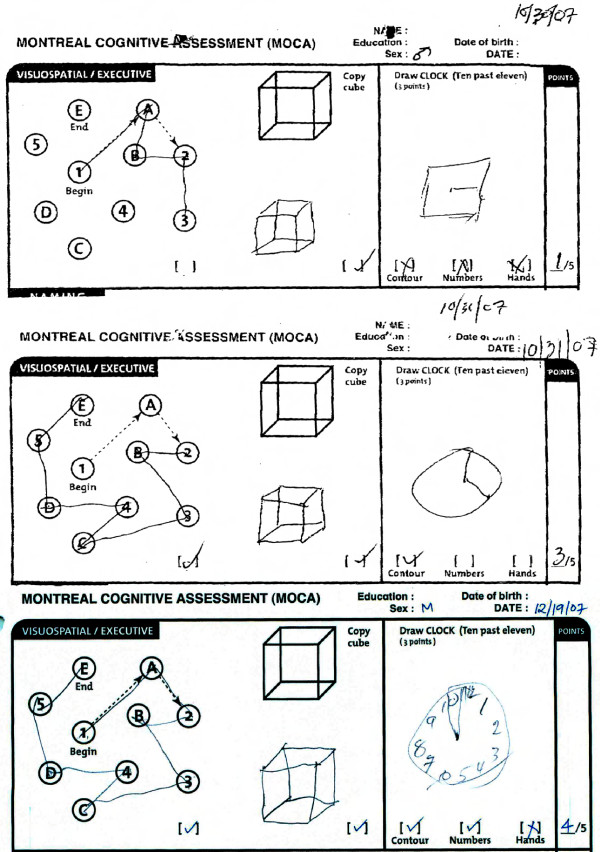
**Rapid and sustained improvement in Visuospatial/Executive function following perispinal etanercept documented by the Montreal Cognitive Assessment**. The top panel depicts the first three tasks from the Montreal Cognitive Assessment completed by the patient one day prior to perispinal etanercept administration. The middle panel depicts the patient's results two hours after perispinal etanercept administration, showing correct completion of the alternating trail making task documenting improved executive function, and improvement in depiction of a clock face. The bottom panel depicts the patient's results at seven weeks, fourteen days after receiving his previous dose of perispinal etanercept, showing further improvement in his drawing of the clock face, with numerals added, and persistence in improvement in completion of the trail making task. (The instruction for completion of the alternating trail making task are as follows: *Please draw a line, going from a number to a letter in ascending order. Begin here *[point to (1)] *and draw a line from 1 then to A then to 2 and so on. End here *[point to (E)]).

The patient was able to copy a three-dimensional cube, but when asked to draw a clock with the hands placed at ten minutes after 11, he drew a square without numbers and one line for the hour or minute hand [see Figure 1]. He could not do the alternating trail making task and was clearly overwhelmed by the task [see Figure 1]. He was able to pantomime four out of eight finger constructions of increasing complexity.

On memory testing the patient could not recall the name of physician HG after 90 seconds despite repetitive introductions on at least four occasions throughout the examination. It took three trials for the patient to register five words and 90 seconds later the patient could not retrieve any of the words even with categorical cueing. When given multiple choice cueing he was able to retrieve two words.

On an A vigilance task, screening for sustained attention, the patient made four errors of omission. He was able to list five digits in a forward fashion, but could not list three digits in a reverse fashion.

The patient could not perform simple calculations and could not do serial sevens. When asked to add 29 plus 11 after a marked latency he said 31.

Abstract concepts such as a how a train and a bicycle, a watch and a ruler, or music and painting were similar could not be expressed by the patient.

On the Montreal Cognitive Assessment test (MOCA) [39] the patient scored seven out of 30 possible points, consistent with a moderate to severe cortical dementia.

The patient met the NINCDS-ADRDA Criteria for probable AD[40], and, in addition, met the Diagnostic and Statistical Manual of Mental Disorders, Fourth Edition (DSM-IV) criteria for AD[41].

### Laboratory results

MRI examination in March 2006 showed age-related diffuse central and cortical atrophy. In November 2006 FDG positron emission tomography brain scan at UCLA showed moderate to severe cortical atrophy, with atrophy-associated global cortical hypometabolism and superimposed biparietal, left temporal and posterior cingulate cortex hypometabolism, read as consistent with Alzheimer's disease.

Laboratory examination prior to treatment revealed a stable but borderline elevated blood urea nitrogen and creatinine, but was otherwise unremarkable, including chest x-ray which was unremarkable, normal thyroid function, normal liver function tests, normal complete blood count, normal serum glucose and electrolytes, normal prostate specific antigen, cholesterol, triglycerides, serum folic acid and vitamin B12 levels, non-reactive RPR and non-reactive FTA-ABS.

### Treatment

Written informed consent for administration of etanercept was obtained from the patient and his wife after the potential risks of etanercept, including infection, cytopenias, possible increased risk of lymphoma and demyelinating disease, death, eye inflammation, and congestive heart failure; and alternative treatment approaches were enumerated.

Immediately prior to etanercept administration the patient was questioned by author ET. He could not state the year nor could he name the state.

Twenty-five mg of etanercept in 1 cc of sterile water was administered by posterior cervical interspinous injection in the midline with a 27 gauge needle at the C6–7 interspace followed by Trendelenburg positioning with the head dependent for five minutes, as previously described, to effect entry of etanercept into the cerebrospinal venous system[21,27]. The patient had no difficulty with this positioning and resumed sitting posture without incident.

Ten minutes after dosing the patient was reexamined. He was noticeably calmer, less frustrated, and more attentive. He was able to correctly identify the state as California, and he identified the year as 2006. His responses to questioning seemed less effortful and more rapid, with less latency. He left for author HG's office for further testing.

### Neurologic examination two hours after etanercept treatment

The patient was retested by author HG two hours after etanercept administration. He could not recall being in author ET's office earlier in the day, but could recall HG's name. He was now oriented to month, day of week, and place, and could again correctly name the state as California, which he could not do prior to perispinal etanercept administration. He was off on the calendar date by two days, and year by one year. Throughout testing he appeared more aware of his deficient performance.

The patient's ability to name pictures was markedly improved, correctly naming nine out of the first ten pictures on the Boston Naming Test short form, requiring phonemic cueing for three of the items (squid, bench, and hammock), a marked improvement from the day prior when he could only name one of the ten presented pictures and used marked circumlocution. On the verbal fluency FAS test he was able to list eight words that started with the letter F in 60 seconds and made only one perseveratory response. On a categories semantic task he was able to list five animals in 60 seconds.

On visuospatial functioning, the patient could copy a three-dimensional cube, and when asked to draw a clock he drew a circle with an hour and minute hand [see Figure 1]. When given a modified trails B test the patient was clearly able to sequence and complete the task, alternating letters and numbers accurately [see Figure 1]. He was able to pantomime seven out of eight finger constructions of increasing complexity.

On memory testing the patient could not recall any of five memoranda after five minutes even with categorical cueing. With multiple-choice cueing he could recall one out of five memoranda. However, as mentioned he could recall the examiner's name without difficulty and without necessity of repetition of introductions throughout the entire examination.

On attentional testing, the patient could accurately list five digits in a forward fashion and three digits in a reverse sequence fashion. On an A vigilance task he made no errors of omission or commission.

On calculations, he could subtract seven from one hundred correctly, but could not perform serial sevens. He could not divide 58 by two, nor could he add 29 plus 11. He could not tell physician HG how many nickels were in a dollar.

On ability to abstract concepts he was able to relate how a train and bicycle were similar in that they both can be used for transportation. When asked how a watch and a ruler were similar he related that they both give information. When asked how music and painting were similar he related "you draw your painting, however music you hear".

Montreal Cognitive Assessment performed two hours following the single dose of perispinal etanercept yielded a score of 15 out of 30 points [see Figure 1].

A collateral interview with the patient's son and wife confirmed their validation of a marked improvement in cognitive abilities following perispinal etanercept administration.

Upon returning to the clinic one week following perispinal etanercept administration for his weekly dose the patient's wife and son confirmed that he had remained markedly clinically improved throughout the week, a fact which was remarked upon by the family [see Additional file 1]. He was noticed to be less reluctant to join in conversation. On re-examination by author ET prior to repeat dosing one week after the initial dose, the patient correctly identified the year, month, season, day of week and state. He appeared to answer with less frustration, and the examiner's impression was that there was reduced latency of response, and his affect seemed improved. On the FAS test for verbal fluency when asked to list all of the words that start with the letter F in 60 seconds he listed 8 words, and named 5 animals in 60 seconds. The patient received a single dose of perispinal etanercept for each of the first five weeks; the next dose was omitted, and the patient returned after seven weeks and was retested. At seven weeks, fourteen days after receiving his last dose of perispinal etanercept, his Montreal Cognitive Assessment score was 14, with persistent improvement in the trail making task, and improved depiction of the clock face [see Figure 1].

## Discussion

The authors' preferred hypothesis to explain the rapid clinical improvement observed centers on the emerging evidence, which suggests that TNF-alpha is of critical importance in the regulation of synaptic transmission in the brain. The authors are led in this direction by the combination of:

1. The extreme rapidity of the effect;

2. The extraordinary potency and selectivity of etanercept as an anti-TNF-alpha agent, due to its biologic nature and molecular structure;

3. The various lines of scientific evidence which have suggested that synaptic dysfunction may be of key importance in the pathogenesis of Alzheimer's disease [28-32];

4. Evidence suggesting that TNF-alpha regulates synaptic transmission in the brain[33-35,42,43]; and,

5. Evidence suggesting that TNF-alpha mediates the synaptic dysfunction underlying cognitive and behavioral impairment produced by both beta-amyloid and beta-amyloid oligomers [36,37].

A weakness of the present study is the fact that all participants, including the examining physicians, were aware of the treatment modality utilized, which could, potentially, bias the results, and a placebo effect cannot be ruled out. However, it should be noted that the clinical, cognitive, and behavioral improvement in the present patient was noted by the patient's family members, family friends, and both authors, and confirmed by the use of several objective measures.

The eight point improvement on the MOCA, from a score of seven to a score of 15, is notable because it exceeds the normal test-retest variation (0.9+/-2.5 points[44]) by more than three standard deviations. The MOCA was designed to measure eight cognitive domains, and is particularly sensitive to changes in executive function[44]. This is illustrated by the patient's results on the trail-making portion of the test, as well as in depicting a clock face [see Figure 1].

Improved verbal fluency, as suggested by this patient's improvement in category naming, animal naming, and Boston Naming results, is a characteristic effect of perispinal etanercept, in the authors' experience. This has been particularly noticeable in patients with probable Alzheimer's disease, such as the present patient, who present with difficulties in word finding, which has included one patient with primary progressive aphasia treated by the authors. Improved behavior, and improvement in affect, as seen in this patient, is another characteristic effect of perispinal etanercept in the authors' experience.

It should also be emphasized that rapid cognitive improvement following perispinal etanercept is not limited to the patient of the present report, but has, in fact, been commonly observed in multiple patients during the authors' now more than three year clinical experience utilizing perispinal etanercept for treatment of probable Alzheimer's disease[20,21]. The rapid response to perispinal etanercept may provide an important clue to the pathophysiologic mechanisms underlying not only Alzheimer's disease, but also other brain disorders involving excess TNF-alpha and cognitive dysfunction, including frontotemporal dementia[45], and traumatic brain injury [46].

Complex problem solving requires intact pre-frontal and anterior cingulate function allowing recall of appropriate and relevant past, present, and future deeds, assigning emotional valence to them and comparing them to the present situation to manage an anticipated sequence of events in a flexible and goal-oriented fashion. Prefrontal neuropathological change of diverse etiologies, including Alzheimer's disease interferes with such complex functions. The assessment of such executive dysfunction is poorly reflected in most standard screening psychometrics. Multiple authors have commented on patients with frontal lobe deficits that perform well on most standardized psychometric tests but have incapacitating disability to function in a real, complex life environment [47,48]. The screening of isolated cognitive domains without screening for executive dysfunction, by simple tests such as the Mini-Mental Status Examination or Cognitive Abilities Screening Instrument, can easily miss critical deficits in executive functioning in patients with involvement of the anterior cingulate and pre-frontal lobes.

Executive dysfunction is one of the chief components of the final common pathway of disabilities in Alzheimer's disease that appear to rapidly improve with perispinal etanercept as documented above. Without quantifying executive function, a clinician or family observation of an improvement in a patient's ability or behavior may improperly be discounted as a subjective interpretation. The Montreal Cognitive Assessment has several screens for executive dysfunction, including a modified Trails B (alternating trail making task) [see Figure 1] that reflect the quantum improvement noted in the present patient, thereby objectifying the patient's family and clinicians' subjective impressions.

Innate immune regulation of brain function is an area of intense current interest [34,42,43,49-51]. The rapid clinical improvement which is characteristic of Alzheimer patients treated with perispinal etanercept and which is described herein provides a new clue for investigating these mechanisms. One suspects that this rapid clinical effect is related to the role of TNF-alpha as a regulator of synaptic mechanisms in the brain, which was first described more than a decade ago[52]. Glia are now known to envelop neuronal synapses in the brain and release molecules, gliotransmitters, which regulate synaptic transmission in those enveloped synapses[42,50]. TNF-alpha is one of only a handful of recognized gliotransmitters[33,34,42,50]. In experimental models, TNF-alpha alters synaptic transmission in rat hippocampal slices, and produces a rapid exocytosis of AMPA receptors in hippocampal pyramidal cells [35,52]. TNF-alpha released by glia, has been demonstrated to control synaptic strength[33,34,53,54].

Synaptic scaling has been suggested to be centrally involved in the synaptic dysfunction occurring in Alzheimer's disease[30,55]. Synaptic scaling involves uniform adjustments in the strength of all synaptic connections for a neuron in response to changes in the neuron's electrical activity[34,53,54]. Synaptic scaling is a homeostatic mechanism which is necessary for the optimal functioning of neural networks [34,53,54]. Synaptic scaling has been demonstrated to be regulated by glial TNF-alpha[34]. These experimental findings, together with the rapid effects of perispinal etanercept shown here, converge to suggest that synaptic dysregulation produced by excess TNF-alpha [5,7-10,12,14,16,18,23] contributes to cognitive and behavioral dysfunction in Alzheimer's disease. Furthermore, our findings suggest that this synaptic dysfunction may, at least in part, be reversible with anatomically targeted anti-TNF-alpha treatment.

Of relevance to the present report is recent evidence that stimulation of a single neuron can cause a change in an animal's behavior[56]. This may be possible because of the massively interconnected nature of the brain: a single cortical pyramidal cell connects to several thousand postsynaptic neurons[56]. In addition, the processes of one astrocyte may make contact with over 100,000 synapses [50]. Processes affecting glial-neuronal interaction may therefore have potential for rapid amplification of their cognitive and behavioral effect.

One may therefore extrapolate from these findings and theorize how perispinal etanercept might have rapid widespread effects.

The authors hypothesize that excess TNF-alpha in Alzheimer's disease[5,7-10,12,14,16,18,23] interferes with the synaptic regulatory functions of TNF-alpha. When TNF-alpha is in a normal physiologic range synaptic scaling is enabled, thereby preserving optimal functioning of the brain's neural network. When TNF-alpha is overexpressed, due to glial activation, it is postulated that the synaptic regulatory activities of TNF-alpha are disturbed. Synaptic dysfunction is hypothesized to result from this dysregulation, which may provide a basis for reduced functional connectivity between brain regions in Alzheimer's disease [57,58]. The rapid effects of perispinal etanercept are hypothesized to be the result of rapid neutralization of excess TNF-alpha, which thereby ameliorates this synaptic dysregulation, allowing normal cross talk between different regions of the brain.

A cytokine concentration-dependent duality of physiologic effect, which the authors hypothesize occurs in TNF-alpha modulation of synaptic function, has been observed with respect to interleukin-1(IL-1) regulation of long-term potentiation in hippocampal slices[59]. This data suggests that IL-1 is required for LTP under physiological conditions, but at higher doses, as may be encountered in certain pathological conditions, IL-1 inhibits LTP[59]. The authors suggest that a similar duality occurs with respect to TNF-alpha in Alzheimer's disease.

It is speculated, on the basis of the clinical results observed and the studies cited above, that optimal synaptic function in the human brain requires that TNF-alpha remain within a physiologic range; perhaps analogous to the necessity to maintain serum calcium in a narrow physiologic range to preserve optimal neuronal function. The positive clinical effects the authors have observed using perispinal etanercept for chronic treatment of Alzheimer's disease on an open-label basis [20,21], now for a period exceeding three years, suggest that maintenance anti-TNF-alpha treatment may have prolonged beneficial effects. It is hoped that future studies may clarify if maintenance perispinal etanercept treatment will help to maintain or restore TNF-alpha homeostasis in the brain and/or cerebrospinal fluid in patients with Alzheimer's disease.

Excess TNF-alpha now appears to preliminarily satisfy the neurologic equivalent of Koch's postulates [60] with respect to being an important component of the pathophysiology of Alzheimer's disease, in that scientific evidence of the following has been published:

1. Excess TNF-alpha, at a level 25 times higher than controls, has been documented in the cerebrospinal fluid of patients with Alzheimer's disease(AD); and elevated CSF TNF-alpha correlated with progression from mild cognitive impairment to AD [7-9];

2. Examination of TNF-alpha polymorphisms in population studies has provided evidence that genetic polymorphisms associated with increased TNF-alpha production are associated with increased AD risk and decreased age of AD onset [10,16,18];

3. Increased spontaneous production of TNF-alpha by peripheral blood mononuclear cells was associated with increased AD risk in a population of patients followed as part of the Framingham study[23];

4. Multiple basic science studies suggest the involvement of TNF-alpha in neuroinflammatory mechanisms which may contribute to AD pathogenesis [5,6,11,15,17,19,24,25,33-37,51,61-72];

5. TNF-alpha is a gliotransmitter[33-35,42,50,51];

6. Glial TNF-alpha may regulate synaptic mechanisms involving synaptic function in neural networks[33-35,50-54];

7. Disruption of memory mechanisms by beta-amyloid and beta-amyloid oligomers is mediated by TNF-alpha [36,37]; and

8. Pilot evidence supporting the efficacy of anatomically targeted[27] anti-TNF-alpha treatment for Alzheimer's disease has recently been published[20,21].

The case presented here provides clinical evidence of a rapidly reversible, TNF-alpha-related component to the cognitive dysfunction present in Alzheimer's disease. This reversible mechanism necessarily precedes irreversible neuronal structural damage, somewhat analogous to the reversible ischemic penumbra that surrounds cerebrovascular infarction. The rapid treatment response is consistent with emerging evidence suggesting that glial-derived TNF-alpha may regulate brain synaptic mechanisms. These mechanisms are worthy of further investigation, and may lead to earlier therapeutic intervention which may have the potential to favorably affect the natural history of Alzheimer's disease.

## Abbreviations

TNF-alpha: Tumor necrosis factor-alpha; 

AD: Alzheimer's disease; 

HG: Hyman Gross, MD; 

ET: Edward Tobinick, MD; 

MRI: Magnetic resonance imaging; 

PET: Positron emission tomography; 

FDG: Fluorodeoxyglucose. 

NINCDS-ADRDA: National Institute of Neurological Communicative Disorders and Stroke-Alzheimer's disease and Related Disorders Association;

MOCA: Montreal Cognitive Assessment;

IL-1: Interleukin-1;

LTP: Long-term potentiation.

## Competing interests

Author Hyman Gross MD has no competing interests. Author Edward Tobinick owns stock in Amgen, the manufacturer of etanercept, and has multiple issued and pending patents, assigned to TACT IP LLC, which describe the parenteral and perispinal use of etanercept for the treatment of Alzheimer's disease and other neurological disorders, including, but not limited to, U.S. patents 6015557, 6177077, 6419934, 6419944, 6537549, 6982089, 7214658 and Australian patent 758523.

## Authors' contributions

Both authors take full responsibility for the entire content of the article, and give approval for the final submitted version of the article; Edward Tobinick was the principal author. Hyman Gross performed the neurological examinations before and after perispinal etanercept treatment, contributed to the discussion section, and reviewed the entire manuscript.

## Supplementary Material

Additional file 1 Quicktime movie. video depicting family's description of change in patient following perispinal etanercept treatment. Click here for file
